# Exposure to essential and non-essential trace elements and risks of congenital heart defects: A narrative review

**DOI:** 10.3389/fnut.2023.1121826

**Published:** 2023-03-14

**Authors:** Yipu Liang, Zijian Pan, Mingzheng Zhu, Ruonan Gao, Yijue Wang, Yijuan Cheng, Nannan Zhang

**Affiliations:** ^1^National Center for Birth Defect Monitoring, Key Laboratory of Birth Defects and Related Diseases of Women and Children, Ministry of Education, West China Second University Hospital, Sichuan University, Chengdu, China; ^2^West China Hospital of Stomatology, Sichuan University, Chengdu, China; ^3^West China Hospital, Sichuan University, Chengdu, China; ^4^West China School of Pharmacy, Sichuan University, Chengdu, China

**Keywords:** congenital heart defects, cardiac development, trace elements, heavy metals, environmental hazards

## Abstract

Congenital heart defects (CHDs) are congenital abnormalities involving the gross structures of the heart and large blood vessels. Environmental factors, genetic factors and their interactions may contribute to the pathogenesis of CHDs. Generally, trace elements can be classified into essential trace elements and non-essential trace elements. Essential trace elements such as copper (Cu), zinc (Zn), iron (Fe), selenium (Se), and manganese (Mn) play important roles in human biological functions such as metabolic function, oxidative stress regulation, and embryonic development. Non-essential trace elements such as cadmium (Cd), arsenic (As), lead (Pb), nickle (Ni), barium (Ba), chromium (Cr) and mercury (Hg) are harmful to health even at low concentrations. Recent studies have revealed the potential involvement of these trace elements in the pathogenesis of CHDs. In this review, we summarized current studies exploring exposure to essential and non-essential trace elements and risks of CHDs, in order to provide further insights for the pathogenesis and prevention of CHDs.

## 1. Introduction

Congenital heart defects (CHDs) are defined as congenital abnormalities involving the gross structures of the heart and large blood vessels (1). As one of the most common congenital malformations and the leading cause to childhood death, CHDs affect around 8–10 per 1,000 live births in the world (2). The mortality of CHDs presents an overall trend of decline worldwide since 1990, due to the development of diagnostics and cardiac surgery (3). However, the economic and health burden of CHDs are still heavy, especially in low-and lower-middle-income countries (3–5).

CHDs can be generally classified into four major subtypes according to the pathophysiological mechanisms of CHDs, including left-to right circulatory shunts, right-to-left circulatory shunts, right ventricular outflow tract obstruction (RVOTO), and left ventricular outflow tract obstruction (LVOTO) (6). Left-to-right circulatory shunts refer to the situation in which the oxygenated blood is shunted into chambers or vessels that carry deoxygenated blood due to the abnormal connection between the left and right side of the heart or between aortic and pulmonary artery (7). Common CHDs types of left-to-right circulatory shunts including atrial septal defect (ASD), ventricular septal defect (VSD), and patent ductus arteriosus (PDA) (7). These diseases rarely show the symptoms of cyanosis at early stage, but can ultimately lead to congestive heart failure and Eisenmenger syndrome due to excess flow (8). Right-to-left circulatory shunts such as tetralogy of Fallot (TOF) and transposition of the great arteries (TGA), are characterized by the direct shunt of desaturated venous blood into systemic circulation, leading to persistent cyanosis (9). RVOTO often involve a defect in the pulmonary valve, the infundibulum, or branches of the pulmonary arteries (10). Pulmonary valve stenosis (PVS) and double chambered right ventricle (DCRV) are common types of RVOTO, and can lead to severe hypoxia (11). LVOTO refers to the obstruction of the blood ejecting from left ventricle to the aorta, such as subaortic stenosis (SAS), bicuspid aortic valve (BAV), supravalvular aortic stenosis (SVAS), coarctation of the aorta (CoA), and hypertrophic cardiomyopathy (HCM) (12).

Although not fully clarified yet, environmental factors, genetic factors and their interactions may contribute to the pathogenesis of CHDs (13–15). It is estimated that around 20% of CHDs cases can be attributed to genetic syndromes and teratogens, and the remaining 80% of cases are considered to be multifactorial, caused by combinations of genetic and environmental factors (16). Different kinds of maternal conditions, including maternal illness, malnutrition, pollutants and toxic exposure can lead to increased risks of CHDs (14). Conversely, maternal multivitamin supplements (including folic acid) may reduce risks of CHDs in offspring (17–19). In addition, a potential beneficial role of maternal residential greenness on CHDs has been recently identified (20).

Trace elements are another potential factors that related to the occurrence of CHDs. Some essential trace elements such as copper (Cu), zinc (Zn), and iron (Fe) are essential for human body, and these essential heavy metals may protect the cardiac development (21–27). However, these elements can be toxic at higher concentrations, and studies have demonstrated that excessive exposure to Zn and Cu and may also induce CHDs (28, 29). While non-essential toxic elements such as cadmium (Cd), arsenic (As), lead (Pb), nickel (Ni), barium (Ba), chromium (Cr) and mercury (Hg) are harmful to health even at low concentrations, and maternal exposure to these elements may increase the risks of CHDs (30–34). There are also some trace elements (e.g., selenium (Se), manganese (Mn)) whose effects on cardiac development are still equivocal, which will be discussed in the following text.

The role and pathogenic mechanisms of essential and non-essential trace elements in CHDs have not been reviewed previously. Consequently, the presented review aims to summarize the potential relationship between several trace elements including Cu, Zn, Fe, Se, Mn, Cd, As, Pb, Ni, Ba, Cr, Hg and CHDs, and to provide further insights for the pathogenesis and prevention of CHDs.

## 2. Essential trace elements and congenital heart diseases

### 2.1. Copper and congenital heart diseases

Cu is an essential trace element for bodies. Besides playing important roles in several biological activities including electron transfer and scavenging free radical (35), Cu also involves in embryonic development (36). In Menkes disease, an X-linked recessive genetic disease characterized by Cu metabolism disorder, a higher CHDs prevalence (4.2%) was found as compared with general populations (about 1%), suggesting a possible involvement of Cu dysregulation in cardiac development (37).

**Figure 1:**
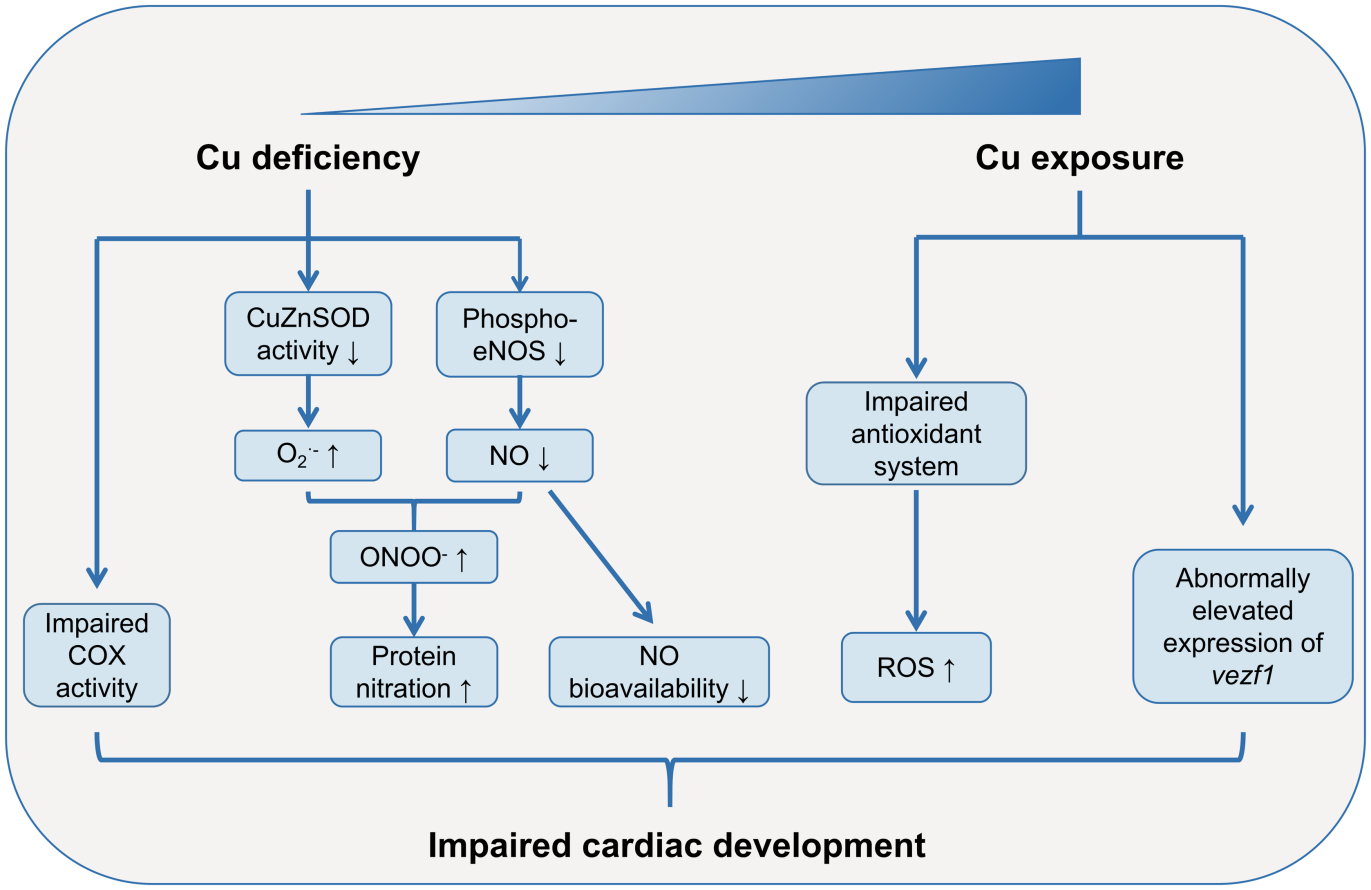
Role of Cu in cardiac development. Both Cu deficiency and excessive Cu exposure may lead to abnormal cardiac development. Cu deficiency dampens the activity of COX in cardiac mitochondria. Cu deficiency impairs anti-oxidant defense system, resulting in increased ROS levels in embryonic heart. Moreover, the large consumption of NO and decreased phosphorylation levels of eNOS due to Cu deficiency lead to low bioavailability of NO during heart development. Excessive Cu exposure can also impairs anti-oxidant defense system, leading to increased ROS levels in embryonic heart. Cu exposure also alters the expression patterns of development-related gene such as *vezf1*, which may lead to abnormal cardiac development. COX, cytochrome-c oxidase; Cu, copper; CuZnSOD, Cu-Zn superoxide dismutase; eNOS, endothelial nitric oxide (NO) synthase; NO, nitric oxide; ROS, reactive oxygen species; *vezf1*, vascular endothelial zinc finger 1.

Excessive Cu exposure is associated with increased risks of CHDs (Figure 1). A case–control study showed that the offspring of mothers with hair Cu levels ≥17.77 μg/g had a 6-fold risks of CHDs, as compared with that of mothers with hair levels of 5.61–17.77 μg/g (OR = 5.70, 95% CI: 2.58, 12.61) (38). Similarly, a specific pattern of cardiac malformation syndrome including double-outlet right ventricle, pulmonary hypoplasia and ventricular spetal defect has been detected in offspring of hamsters that exposed to excessive Cu during pregnancy (29, 39). The abnormal morphological structures and impaired physiological functions of embryonic heart induced by Cu exposure have also been found in several model organisms such as red sea bream (*Pagrus major*) (40), rare minnow (*Gobiocypris rarus*) (28), and marine medaka (*Oryzias melastigma*) (41). Although the specific mechanisms are largely unknown, it has been proposed that impaired anti-oxidant system and altered development-related gene expression patterns may be responsible for Cu-induced cardiac malformation (28).

However, Cu is also an essential trace element for cardiac development, which has been studied in several animal models with Cu deficiency (Figure 1). Uriu-Adams’s group cultured rodent embryos from dams fed with Cu-deficient diet in Cu-deficient media, and found high risks of heart abnormalities in these embryos (26, 35, 42). Subsequent studies revealed an impaired anti-oxidant defense system and increased reactive oxygen species (ROS) levels in these Cu-deficient embryos, and supplementation of anti-oxidant agents such as glutathione peroxidase (GPx) and Cu-Zn superoxide dismutase (CuZnSOD) rescued the heart abnormalities (42). The increased ROS levels induced by Cu-deficiency can be partially explained by the dysfunction of cytochrome-c oxidase (COX), the terminal enzyme in the respiratory chain (43). The deficiency of Cu, the pivotal component of COX, led to an impaired COX activity, which could result in leakage of electrons and reaction intermediates and elevated ROS levels in mitochondria (42, 44). Similarly, in offspring of rat dams fed with Cu-deficient diet, the COX subunit 1 (COX-1) and COX-4 activities were dampened in the late postnatal development in cardiac mitochondria (45). Another possible mechanisms on Cu-deficiency-induced heart abnormalities comes from the increased protein nitration and low bioavailability of nitric oxide (NO) (26, 35). Under the circumstance of Cu deficiency, the elevated O_2_^•-^ reacts with NO rapidly to form peroxynitrite (ONOO^−^), leading to increased protein nitration and dysregulated protein functions (35). The large consumption of NO results in a decreased NO bioavailability, which may induce cardiac abnormalities (26). Moreover, the decreased phosphorylation levels of endothelial NO synthase (eNOS) at Ser1177 in Cu-deficient embryos may further exaggerate the low bioavailability of NO (26).

Cu can pass through the placental barrier through the active transport of two Cu-ATPases (ATP7A and ATP7B), therefore, the fetus may also be affected by maternal Cu status (46). However, in a recent case–control study conducted by Yang et al., no association was detected between dietary or supplemental Cu intake in mothers during pregnancy and CHDs occurrence in offspring (47). The results may be interpreted that few pregnant women in the study population had deficient or excessive Cu status. Therefore, it is still unclear whether it is necessary to supplement Cu in Cu-deficient pregnant women in order to prevent CHDs in offspring.

### 2.2. Zinc and congenital heart diseases

Zinc (Zn) is an essential trace element for human, and exerts important roles in regulating immunity and maintaining male reproductive function (48, 49). Most importantly, Zn is also closely associated with cardiac development.

Results from epidemiological studies regarding Zn and CHDs seem to be equivocal, though limited evidence show potential benefit of Zn on heart development. For instance, higher serum Zn levels were associated with decreased odds of isolated ventricular septum defects (VSDs) in children (50). A recent study evaluated the association between maternal dietary or supplementary Zn intake during pregnancy and CHDs risks in offspring (47). The results showed that higher intake of Zn was associated decreased risks of CHDs in offspring. In addition, mothers whose total Zn intake during pregnancy reached the recommended nutrient intakes (RNIs) (i.e., 9.5 mg/d) had lower risks of CHDs in offspring, as compared with those did not reach. These results were consistent with a previous study showed that Zn supplementation in mice rescued cardiac malformation induced by diabetes through modulating oxidative stress (25). However, some studies did not found similar results. A study showed that serum Zn concentrations in CHDs fetus and their mothers were significantly higher than those in controls (51). Several studies demonstrated that no significant difference of maternal Zn levels existed between CHDs and controls (38, 52). Sadoh et al. measured serum Zn levels in 41 children with CHDs and 41 children without CHDs, but found no significant difference between the two groups (53).

**Figure 2:**
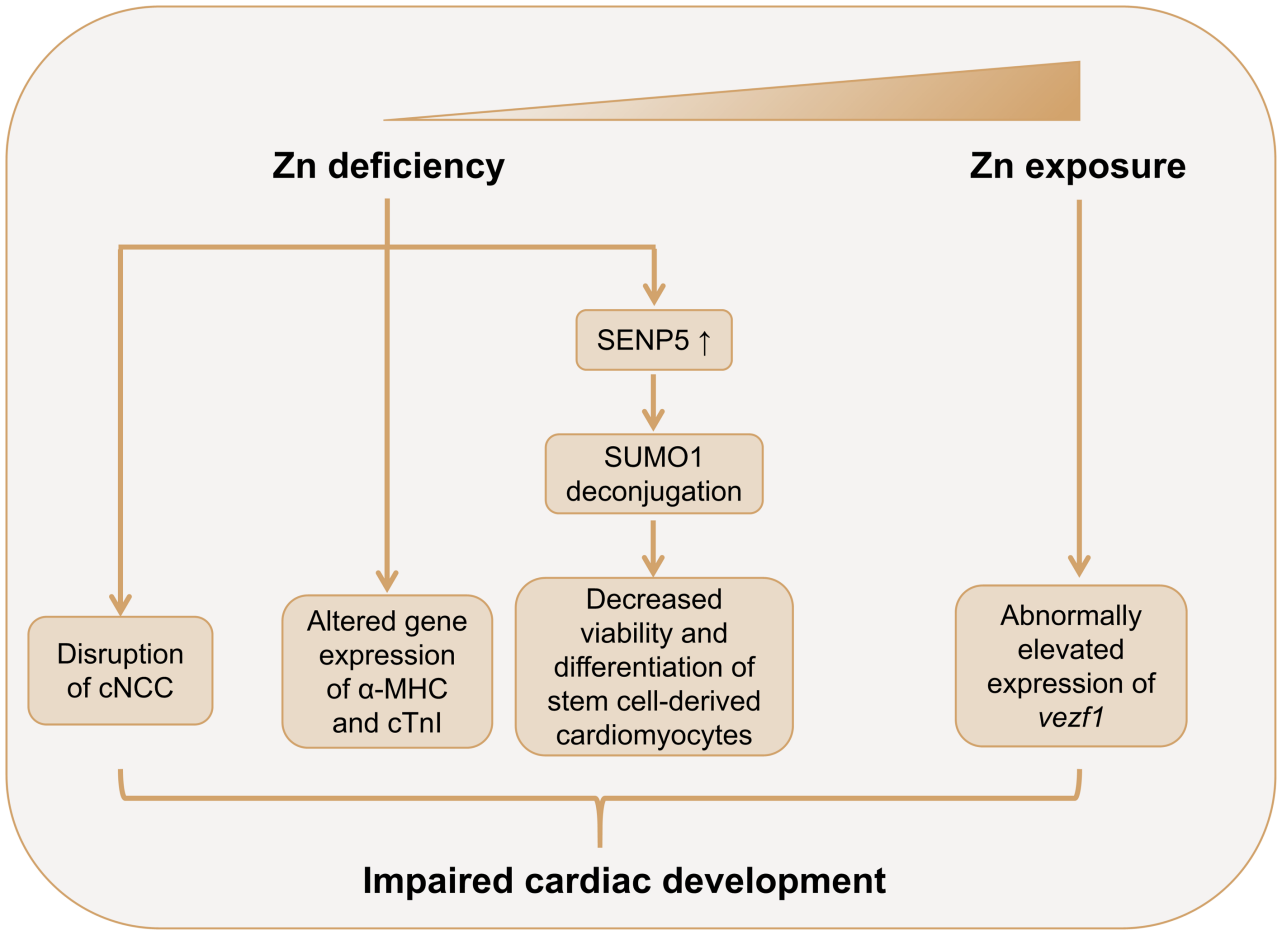
Role of Zn in cardiac development. Both Zn deficiency and excessive Zn exposure may lead to abnormal cardiac development. Zn deficiency leads to abnormal amount and distribution of cNCC in the embryonic heart. Zn deficiency alters the gene expression patterns of α-MHC and cTnI in the fetus heart. Zn deficiency also promotes SENP5 overexpression, which deconjugates SUMO1, leading to decreased cell viability and inhibited differentiation of stem cell-derived cardiomyocytes. Zn exposure may induce cardiac abnormalities though promoting the expression of *vezf1* in embryonic heart. α-MHC, α-myosin heavy chain; cTnI, cardiac troponin I; cNCC, cardiac neural crest cell; NO, nitric oxide; SENP-5, small ubiquitin-related modifier protein (SUMO)-specific protease 5; SUMO-1, small ubiquitin-related modifier protein 1; *vezf1*, vascular endothelial zinc finger 1; Zn, zinc.

However, several animal studies showed a pivotal role of Zn in embryonic heart development (Figure 2). In 1966, Hurley et al. demonstrated a high incidence of abnormalities in the embryonic hearts of Zn-deficient rats (54). Further studies have illustrated that cardiac malformations induced by Zn deficiency were mainly involved in the great vessels, the outflow tract and the development of the atrium and ventricle, which may possibly due to the anomalous distribution, amount, and function of cardiac neural crest cells (cNCC) during embryonic development (55, 56). Indeed, rat cNCC cells cultured in Zn-deficient media exhibited decreased cell viability, and elevated oxidative stress levels and active caspase-3 expressions (57). Another study used connexin-43 (Cx43) and HNK-1 as biomarkers of cNCC and found abnormal amount and distribution of Cx43 and HNK-1 in the embryonic hearts of Zn-deficient rats (56). In addition, the gene expression patterns of α-myosin heavy chain (α-MHC) and cardiac troponin I (cTnI), both of which are important in embryonic cardiac development, were significantly altered in the Zn-deficient rat fetus hearts (55). Zn deficiency also decreased expression levels of metallothionein-1 (MT-1) and zinc transporter-1 (ZnT-1) in rat placentas, which may contribute to the cardiac malformation in Zn-deficient fetus, although the direct evidence is still lacking (58). Zn deficiency-induced cardiac abnormalities is also mediated by dysregulated sumoylation and desumoylation during heart development (41). Zn-deficient fetus demonstrated decreased small ubiquitin-related modifier protein (SUMO)-1 levels and increased SUMO-specific protease (SENP)-5 levels in the embryonic hearts at embryonic day (E) 10.5. Further *in vitro* analysis showed that Zn deficiency induced human induced pluripotent stem cells (hiPSC)-derived cardiomyocytes (hiPSC-CMs) apoptosis, and inhibited cell viability and differentiation, and these adverse effects were mediated through SENP-5 overexpression.

Few studies have investigated the cardiac teratogenicity of excessive Zn (Figure 2). Zhu et al.’s study showed that Zn exposure induced cardiac morphological abnormalities and cardiac dysfunction in embryonic rare minnow (*Gobiocypris rarus*) (28). The study also detected increased expression of *vezf1*, a pivotal regulator in embryonic heart and vascular development, in Zn exposure embryos, which may partially explain the cardiac teratogenicity of Zn.

### 2.3. Iron and congenital heart diseases

High prevalence of Fe deficiency has been evident in patients with cyanotic CHDs, which may be explained by the increased Fe consumption due to excessive erythropoiesis in response to hypoxia (59–61). However, the roles of iron in the embryonic heart development as well as the pathophysiology of CHDs are still largely unknown.

A case–control study conducted by Yang et al. showed that low levels of Fe intake, Fe supplementation, and Fe status in mothers were associated with increased risks of CHDs in offspring (27). Similarly, animal studies also found that Fe deficiency increased risks of cardiac abnormalities. In 2006, Andersen et al. found that cultured rat embryos with Fe deficiency showed poor yolk sac circulation and decreased heart size (62). A recent study further unveiled the essential role of Fe in cardiac development (63). The results showed that maternal Fe deficiency led to increased risks of ventricular septal defects (VSDs), atrioventricular septal defects (AVSD), thin ventricular myocardium in embryos. These CHDs phenotypes in Fe deficient embryos were associated with malrotation of the cardiac outflow tract (OFT), abnormal cardiac cushions, as well as abnormal aortic arch. Further analysis showed that Fe deficiency resulted in elevated retinoic acid (RA) signaling in second heart field (SHF) and OFT, leading to ectopic activation of cardiac transcription factor GATA4 and premature differentiation of SHF cardiac progenitor cells. Most importantly, these CHDs phenotypes can be rescued by RA signaling abrogation or dietary supplementation of Fe during mid-gestation.

Conversely, a recent case–control study demonstrated that high maternal hair Fe concentrations (≥52.95 μg/g) were associated with 2.87-fold risks of fetus CHDs, as compared with pregnant mothers with hair Fe of 43.15–52.95 μg/g (aOR = 2.87, 95% CI: 1.54, 5.37) (64). These results suggest a potential cardiac teratogenic effect of excessive Fe. Moreover, it is also reasonable to deduce that excessive Fe supplementation may increase the risks of fetus CHDs in pregnant women, though direct evidence is still lacking. However, other biomarkers that reflex body Fe status such as haemoglobin and ferritin were not evaluated in the same study.

### 2.4. Other essential trace elements and congenital heart diseases

Se plays important roles in multiple biological processes such as antioxidant defense, cellular signaling, and protein folding (65). Se deficiency has been closely correlated with cardiovascular diseases, including Keshan disease, myocardial infarction, and coronary heart disease (66). However, the role of Se in cardiac development and CHDs is still largely unknown. A case–control study conducted by Ou et al. showed that maternal blood Se collected during middle and late gestation was significantly lower in CHDs infant compared with controls (67). After adjusting several confounding factors, the authors demonstrated that Se exposure >199.67 μg/l was associated with 75% decreased risks of CHDs (adjusted odds ratio (aOR) = 0.25, 95% CI: 0.08, 0.77), compared with Se levels <178.12 μg/L. Similarly, a recent study found that both dietary and supplement intakes during pregnancy were inversely associated with lower risks of CHDs in offspring (47). However, Guo et al. utilized hair to assess Se exposure in pregnant women, and found that high Se exposure was associated with increased risks of CHDs in offspring (68). One possible explanation is the U-shape effect of Se on human health, and the specific role of Se on CHDs may dependent on baseline Se status (69, 70).

Mn is an essential trace metal that implicates in various biological functions such as anti-oxidative stress, metabolic processes, and regulating endocrine (71). However, both Mn deficiency and excessive Mn exposure can lead to increased ROS as well as neurological impairments (72). An epidemiological study showed that mothers with hair Mn >3.01 μg/g has increased risks of CHDs in fetus, as compared with mothers with hair Mn of 0.11–3.01 μg/g (aOR = 2.68, 95% CI: 1.44, 4.99) (64). Conversely, Zhu et al.’s study showed that maternal dietary supplementation of organic Mn reversed oxidative stress and apoptosis induced by heat stress in chick embryonic hearts (73). However, the study did not research the alterations of morphology or functions of embryonic hearts.

## 3. Non-essential trace elements and congenital heart diseases

### 3.1. Cadmium and congenital heart diseases

Cd is a toxic non-essential metal that can result in various types of cancer, osteoporosis, as well as liver and renal diseases (30). Moreover, the teratogenic effect of Cd during cardiac development has been evident. A case–control study evaluated maternal hair Cd levels and CHDs occurrence in offspring showed that high maternal Cd levels (≥ 25.85 ng/g) were associated with a 1.96-fold increased risks of CHDs as compared with low Cd levels (≤7.23 ng/g, aOR = 1.96, 95% CI: 1.24, 3.09) (74). Similarly, in zebrafish (*Danio rerio*) embryos exposed to 10 μM CdCl_2_, some larvae showed hypertrophy of the ventricle and pericardium (75). In addition, Cd exposure during embryos also affected cardiovascular physiology in zebrafish, as evident in reduced heart rate and abnormally elevated heart contractility (75). In chick embryos, Cd exposure increased the myocardial tissue area of the right ventricle, though scarcely gross cardiac malformation was detected (75). Further mechanical analysis showed that these pathological changes were associated with increased cell proliferation and upregulated expression of cell circle related genes such as *Cdk1*, *Cdk6*, *CycA*, *CycD*, and CycE. Since right ventricular hypertrophy has been identified as an independent risk factor of heart failure and cardiovascular death (76), it is speculated that Cd exposure during cardiac development may increased risks of cardiovascular diseases in adulthood. Subsequent study conducted by Hudson et al. may partially confirm this hypothesis, which showed that maternal Cd exposure in mice increased heart weight at birth as well as risks of hypertension in adulthood in offspring (77). However, it is needed to point out that Cd can hardly be detected in offspring of mothers that exposed to Cd, which may be explained by the presence of placental barrier (77). Further study demonstrated that the cardiac alterations may be secondary to the altered essential trace element profiles induced by maternal Cd exposure (77).

### 3.2. Arsenic and congenital heart diseases

As is a toxic metalloid that naturally occurred *via* geogenic processes in the aquifer (78). It is estimated that 200 million people are exposed to high levels of As in the world, which has raised public health concerns (31). Epidemiological studies associated maternal exposure to As in drinking water with increased risks of CHDs in offspring (79–81). Moreover, a case–control study evaluating maternal hair As levels and CHDs in fetus also showed similar results (74). Experimental studies also confirmed that As exposure may affect cardiac development as well as heart function in embryos. Li et al. assessed the developmental toxicity of As utilizing zebrafish (*Danio rerio*) embryos, and the results showed that As exposure significantly decreased the amount of myocardium in the ventricle, and delayed cardiac looping (82). As exposure also dampened heart rate in the zebrafish embryos in time-and dose-dependent manners. Hematoxylin and eosin (HE) staining conducted by the same group also showed increased pericardial cavity as well as elongated atrium and ventricle in As exposed zebrafish embryos, and these effects are potentially mediated *via* suppressed expression of Dvr1, the pivotal regulator of dorsal mesendoderm activity (83). Maternal As exposure in rats increased the risks of CHDs (especially ventricular septal defects (VSDs) and atrial septal defects (ASDs)) in rats, and these effects could be reversed by folic acid supplementation (84). A series studies conducted by Camenisch et al. showed that As and its toxic metabolite (i.e., monomethylarsonous acid) can impair epithelial-mesenchymal transition (EMT) during cardiac development, potentially through affecting TGF-β/Smad signalings (85–87).

### 3.3. Lead and congenital heart diseases

Pb is a toxic element which shows deleterious systematic effects on human body, and its exposure remains an important public health problem (88). It has been reported that Pb exposure is associated with increased risks of cardiovascular diseases such as hypertension and atherosclerosis (89). In particular, prenatal Pb exposure in pregnant woman may affect cardiac development in offspring. For example, a case–control study conducted by Salehi et al. showed significantly elevated blood Pb concentrations in mothers of children with CHDs (90). In another case–control study, Wang et al. evaluated maternal plasma Pb levels in 303 CHD cases and 303 controls, and found that each unit concentration of Pb was associated with a 2.74-fold (95%CI: 1.00, 7.57) increased risks of CHDs after adjusting for several potential confounders (91). Consistent with these results, Ou et al. found that high levels of maternal blood Pb (blood Pb >3.04 μg/dl) were associated increased risks of CHDs in fetus, as compared with mothers with low blood Pb levels (<2.15 μg/dL) (aOR = 12.09, 95% CI: 2.81, 51.97) (67). Moreover, a study that utilizing maternal hair Pb as a biomarker of Pb exposure also detected Pb as a harmful factor for CHDs (92). These results showed a significant and positive association between Pb exposure and CHDs, however, there is still a lack of studies that exploring the causal effect of Pb on cardiac development and the underlying mechanisms.

### 3.4. Other non-essential trace elements and congenital heart diseases

Ni is a ubiquitous metal in the earth, and has been associated with allergy and carcinoma in human-beings (30). A previous case–control study conducted by our group first associated maternal Ni exposure with fetus CHDs (93). The results showed that maternal hair Ni >0.7216 ng/mg was associated with 2.672-fold risks of CHDs in offspring as compared with mothers with hair Ni <0.4111 ng/mg (aOR = 2.672, 95% CI: 1.623, 4.399). Laboratory-based studies further support the cardiac teratogenic effect of Ni. In marine medaka (*Oryzias melastigma*) embryos, Ni exposure induced cardiovascular anomalies as well as dysregulated expression of cardiac development-related genes including *ATPase*, *smyd1*, *cox2*, and *bmp4* (94). Similarly, Ni exposure in zebrafish (*Danio rerio*) embryos led to abnormally increased expression of cardiac gene including *gata4* and *nkx2.5* (95). One possible explanation for the cardiac teratogenesis role of Ni is that excessive Ni can impair the electrophysiology of Ni^2+^-sensitive T-type Ca^2+^ channels in the embryonic hearts (96, 97).

Ba is a widespread heavy metal in nature, and its poisoning can lead to impairment of gastrointestinal, cardiovascular, and musculoskeletal systems (98). One potential mechanism is that Ba can inhibit the potassium inward rectifier channels (IRCs), thus affecting all types of muscle (99). Particularly, a case–control study that conducted by our group linked prenatal Ba exposure with CHDs in offspring (100). In this study, we measured maternal hair Ba concentrations in 399 CHDs and 490 controls, and found that Ba exposure was associated with increased risks of CHDs and major subtypes (i.e., septal defects, right ventricular outflow track obstruction, left ventricular outflow track obstruction, and anomalous pulmonary venous return) in offspring in a dose–response manner.

Cr is a heavy metal that may play roles in regulating the metabolism of glucose, lipid, and protein (101). However, there is no convincing evidence that can confidently conclude the essential role of Cr in human beings (102). Conversely, Cr exposure has been associated with multiple health hazards such as allergy, impaired reproductive system, and cancer (103). Cr exposure during pregnancy has been associated with poor fetal biometric parameters such as abdominal circumference and estimated fetal weight, suggesting the embryonic toxicity of Cr (104). Ou et al. explored the association between maternal blood Cr levels during pregnancy and CHDs in offspring, but did not detect significant result (67).

Hg is a toxic heavy metal that is hazardous to human health (105). Long term exposure to a low level Hg also leads to cardiovascular, reproductive, and developmental toxicity (106). A recent case–control study evaluated Hg levels in maternal plasma found that each unit concentration of Hg was associated with 2.88-fold risks of CHDs in offspring (aOR = 2.88, 95% CI: 1.22, 6.77) (91). However, there is still a lack of study that validate the causal effects as well as the specific mechanisms between Hg and cardiac development.

## 4. Conclusion and perspectives

In this review, we critically summarized the potential roles of several essential trace elements (Tables 1, 2) and toxic heavy metal elements (Tables 3, 4) in CHDs. Although a large amount of studies have been conducted to explored this issue, it is still equivocal to reach a consistent conclusion based on the current evidence.

**Table 1:** Summary of epidemiological studies reporting the relationship between essential trace element exposure and CHDs.

Exposure	Assessment method	Collection time	Country	Study design	CHD subtypes	Sample size	Effect size	Adjustment variables	Reference
Cu	Maternal hair Cu assessed by ICP-MS	NA	China	Case–control study	Total CHDs	Case: 212; control: 212	Medium Cu (5.61–17.77 μg/g) as reference High Cu (>17.77 μg/g): 5.70 (2.58–12.61); Low Cu (≤5.61 μg/g): 0.63 (0.15, 2.73)	Maternal age, maternal residence, folic acid supplementation, and previous pregnancy	(38)
Cu	Maternal blood Cu assessed by ICP-MS	Middle to late gestation (17th–40th week)	China	Case–control study	Total CHDs	Case: 112; control: 107	High Cu (>932.23 μg/l) vs. Low Cu (<835.22 μg/l): 0.77 (0.31, 1.89)	Maternal age, parity, education, newborn gender, migrant, folic acid or multivitamin intake, cigarette smoking, maternal pre-pregnancy BMI, and time of sample collection	(67)
Zn	Serum Zn in children assessed by ICP-MS	NA	China	Cross-sectional study	VSDs	Case: 144; control: 144	High Zn (>5.11 μg/l) vs. Low Zn (<3.63 μg/l): 0.03 (0.01, 0.29)	Age, and sex	(50)
Zn	Maternal total Zn intake assessed by questionnaire	During pregnancy	China	Case–control study	Total CHDs	Case: 474; control: 948	Met RNI (≥9.5 mg/d) vs. Below RNI (<9.5 mg/d): 0.56 (0.37, 0.84)	Total energy intake during pregnancy, socio-demographic characteristics (maternal age, residence, education, work, and parity), maternal health-related factors in the first trimester (folate/iron supplements use, passive smoking, medication use, and anemia), and dietary diversity score	(47)
Fe	Maternal total Fe intake assessed by questionnaire	During pregnancy	China	Case–control study	Total CHDs	Case: 474; control: 948	High Fe (>34.13 mg/d) vs. Low Fe (<21.14 μg/l): 0.20 (0.13, 0.32)	Total energy intake during pregnancy, socio-demographic characteristics (maternal age, gestational age, residence, education, occupation, and parity), maternal health-related factors in the first trimester (passive smoking, medication use, and folate supplements use); and further adjusted for iron supplements use in the associations of dietary iron, heme iron, nonheme iron intakes with CHDs	(27)
Fe	Maternal hair Fe assessed by ICP-MS	Middle gestation (24^th^-28^th^ week)	China	Case–control study	Total CHDs	Case: 322; control: 333	High Fe (>52.95 μg/g) vs. Medium Fe (43.15–52.95 μg/g): 2.87 (1.54, 5.37)	Maternal age, gestational age, number of weeks of folic acid taken after pregnancy, maternal residence, and outside iron exposure	(64)
Se	Maternal blood Se assessed by ICP-MS	Middle to late gestation (17^th^-40^th^ week)	China	Case–control study	Total CHDs	Case: 112; control: 107	High Se (>199.67 μg/l) vs. Low Se (<178.12 μg/l): 0.25 (0.08, 0.77)	Maternal age, parity, education, newborn gender, migrant, folic acid or multivitamin intake, cigarette smoking, maternal pre-pregnancy BMI, and time of sample collection	
Se	Maternal total Se intake assessed by questionnaire	During pregnancy	China	Case–control study	Total CHDs	Case: 474; control: 948	Met RNI (≥65 mg/d) vs. Below RNI (<65 mg/d): 0.23 (0.11, 0.49)	Total energy intake during pregnancy, socio-demographic characteristics (maternal age, residence, education, work, and parity), maternal health-related factors in the first trimester (folate/iron supplements use, passive smoking, medication use, and anemia), and dietary diversity score	(47)
Se	Maternal hair Se assessed by ICP-MS	Middle to late gestation (14^th^-40th week)	China	Case–control study	Total CHDs	Case: 378; control: 510	Medium Se (0.423–0.884 ng/mg) as reference High Se (≥0.884 ng/mg): 3.57 (1.90–6.70); Low Se (<5.61 μg/g): 0.92 (0.53–1.59)	Maternal age, gestational age, maternal education, landfill sites or factory distribution, folic acid supplementation, parental smoking, maternal pre-pregnancy BMI, lead and copper concentration in hair	(127)
Mn	Maternal hair Mn assessed by ICP-MS	Middle gestation (24^th^-28^th^ week)	China	Case–control study	Total CHDs	Case: 322; control: 333	Medium Mn (0.11–3.01 μg/g) as reference High Mn (≥3.01 μg/g): 2.68 (1.44–4.99); Low Se (≤0.11 μg/g): 0.90 (0.39–2.08)	Maternal age, gestational age, number of weeks of folic acid taken after pregnancy, maternal residence, and outside Mn exposure	(64)

BMI, body mass index; CHDs, congenital heart diseases; Cu, copper; Fe, iron; ICP-MS, inductively coupled plasma mass spectrometry; Mn, manganese; NA, not available; RNI, recommended nutrient intake; Se, selenium; VSDs, ventricular septal defect; Zn, zinc.

**Table 2:** Summary of laboratory-based studies reporting the relationship between essential trace element exposure and CHDs.

Element	Animal model	Function	Potential mechanism	Reference
Cu	Embryos from Cu-deficient pregnant rats were cultured in Cu-deficient media	Cu deficiency increased risks of CHDs	Cu deficiency increased oxidative stress; Cu deficiency increased protein nitration and decreased NO bioavailability	(26, 35, 42, 45, 128)
Cu	Embryos from pregnant golden hamsters that intraperitoneally injected with copper citrate	Excessive Cu increased risks of CHDs	NA	(129)
Zn	Embryos from Zn-deficient pregnant rats	Zn deficiency increased risks of CHDs	Zn deficiency disrupted expression pattern and function of cardiac neural crest cells in fetus heart; Zn deficiency altered expression pattern of α-MHC and cTnI in fetus heart; Zn deficiency decreased MT-1 and ZnT-1 expression in placentas	(55, 56, 58)
Zn	Embryos from Zn-deficient pregnant mice	Zinc deficiency induced abnormal development of myocardium in fetus	Zn deficiency decreased SUMO-1 levels and increased SENP-5 levels in embryonic hearts	(41)
Zn	Embryos from diabetic mice that intraperitoneally injected with Zn sulfate	Zn supplementation rescued fetal cardiac malformation induced by maternal diabetes	Zn supplementation decreased oxidative stress; increased antioxidants; and salvage MT-1 expressions in fetus heart	(25)
Zn	Rare minnow (*Gobiocypris rarus*) embryos cultured in Cu-containing media	Zn exposure induced cardiac morphological abnormalities and cardiac dysfunction	Zn exposure increased *vezf1* expression	(28)
Fe	Embryos from Zn-deficient pregnant mice	Fe deficiency increased risks of CHDs	Fe deficiency increased RA signaling in SHF and OFT, leading to ectopic activation of GATA4 and premature differentiation of SHF cardiac progenitor cells	(63)

α-MHC, α-myosin heavy chain; CHDs, congenital heart diseases; cTnI, cardiac troponin I; Cu, copper; Fe, iron; Mn, manganese; GATA4, GATA-binding protein 4; MT-1, metallothionein 1; NA, not available; NO, nitric oxide; OFT, outflow tract; RA, retinoic acid; Se, selenium; SENP-5, small ubiquitin-related modifier protein (SUMO)-specific protease 5; SHF, second heart field; SUMO-1, small ubiquitin-related modifier protein 1; vezf1, vascular endothelial zinc finger 1; Zn, zinc; ZnT-1, zinc transporter 1.

**Table 3:** Summary of epidemiological studies reporting the relationship between non-essential trace element exposure and CHDs.

Exposure	Assessment method	Collection time	Country	Study design	CHD subtypes	Sample size	Effect size	Adjustment variables	Reference
Cd	Maternal blood Cd assessed by ICP-MS	Middle to late gestation (17th–40th week)	China	Case–control study	Total CHDs	Case: 112; control: 107	High Cd (>2.13 μg/l) vs. Low Cd (<1.50 μg/l): 1.26 (0.48, 3.31)	Maternal age, parity, education, newborn gender, migrant, folic acid or multivitamin intake, cigarette smoking, maternal pre-pregnancy BMI, and time of sample collection	(67)
Cd	Maternal hair Cd assessed by ICP-MS	Middle to late gestation (14th–40th week)	China	Case–control study	Total CHDs	Case: 339; control: 333	Cd ≤ 7.23 ng/g as reference 7.23–12.95 ng/g: 2.34 (1.46, 3.76); 12.95–25.85 ng/g: 3.61 (2.23, 5.83); ≥25.85 ng/g: 5.62 (3.43, 9.24)	Maternal age, gestational age, folic acid supplement, BMI, paternal smoking and maternal previous pregnancies	(74)
As	As in drinking water from different settlements reported by previous archives	NA	Hungary	Ecological study	Total CHDs	Case: 9734; control: 5880	High As (>10 μg/L) vs. Low As (<10 μg/L): 1.41 (1.28, 1.56)	Maternal age, and child’s gender	(79)
As	As in drinking water from different settlements reported by previous archives	During the year before birth	France	Ecological study	Total CHDs	5,263 children	High As (≥10 μg/L) vs. Low As (<10 μg/L): 0.89 (0.21, 2.48) for boys; 3.66 (1.62, 7.64) for girls	Maternal age, parity, paid employment during pregnancy, size of residential municipality and year of birth	(80)
As	As in drinking water from different settlements reported by previous archives	At 4 weeks of gestational age	Denmark	Cohort study	Total CHDs	1,042,413 liveborn children	As <0.5 μg/L as reference 0.5–0.9 μg/L: 1.13 (1.08, 1.19); 1.0–4.9 μg/L: 1.33 (1.27, 1.39); ≥5 μg/L: 1.42 (1.24, 1.62)	Year of birth, mother’s educational level and ethnicity	
As	Maternal hair As assessed by ICP-MS	Middle to late gestation (14th–40th week)	China	Case–control study	Total CHDs	Case: 339; control: 333	As ≤62.03 ng/g as reference 62.03–85.85 ng/g: 2.34 (1.46, 3.76); 85.85–117.75 ng/g: 3.61 (2.23, 5.83); ≥117.80 ng/g: 5.62 (3.43, 9.24)	Maternal age, gestational age, folic acid supplement, BMI, paternal smoking and maternal previous pregnancies	(74)
Pb	Maternal blood Pb assessed by ICP-MS	During the third trimester	China	Case–control study	Total CHDs	Case: 97; control: 194	High Pb (≥1.93 μg/L) vs. Low As (<1.72 μg/L): 2.052 (1.086–3.879)	Maternal age, education level, family monthly income, employment, maternal BMI, pregnancy hypertension disease, parity, folic acid supplement, and passive smoke	(130)
Pb	Maternal blood Pb assessed by ICP-MS	Middle to late gestation (17th–40th week)	China	Case–control study	Total CHDs	Case: 112; control: 107	High Pb (>3.04 μg/dL) vs. Low Pb (<2.15 μg/dL): 12.09 (2.81, 51.97)	Maternal age, parity, education, newborn gender, migrant, folic acid or multivitamin intake, cigarette smoking, maternal pre-pregnancy BMI, and time of sample collection	(67)
Pb	Maternal plasma Pb assessed by ICP-MS	Middle to late gestation (14th–40th week)	China	Case–control study	Total CHDs	Case: 303; control: 303	2.74 (1.00–7.57)	Maternal pre-pregnancy body mass index, education, occupation, parity, and periconceptional folic acid supplementation	(91)
Pb	Umbilical serum Pb assessed by ICP-MS	After delivery	China	Case–control study	Total CHDs	Case: 97; control: 201	High Pb (>8.26 ng/mL) vs. Low Pb (<6.96 ng/mL): 1.67 (0.88–3.17)	Maternal age, maternal pre-pregnancy BMI, maternal education level, folic acid supplement, and parental smoking	(131)
Ni	Maternal hair Ni assessed by ICP-MS	Middle to late gestation (14^th^-40th week)	China	Case–control study	Total CHDs	Case: 399; control: 490	High Ni (>0.72 ng/mg) vs. Low Ni (<0.41 ng/mg): 2.67 (1.62–4.40)	Maternal age, gestational age, education, folic acid supplement, parental smoking, maternal pre-pregnancy BMI, hair Cd level, hair As levels, and hair Pb level	(93)
Ba	Maternal hair Ba assessed by ICP-MS	NA	China	Case–control study	Total CHDs	Case: 399; control: 490	High Ba (>4.222 ng/mg) vs. Low Ba (<2.610 ng/mg): 7.387 (4.528–12.053)	Maternal age, gestational age, education, the large factory nearby, taking folic acid, parental smoking, and maternal pre-pregnancy BMI	(100)
Cr	Maternal blood Cr assessed by ICP-MS	Middle to late gestation (17th–40th week)	China	Case–control study	Total CHDs	Case: 112; control: 107	High Cr (>2.13 μg/L) vs. Low Pb (<1.50 μg/L): 0.84 (0.36, 1.96)	Maternal age, parity, education, newborn gender, migrant, folic acid or multivitamin intake, cigarette smoking, maternal pre-pregnancy BMI, and time of sample collection	(67)
Hg	Maternal plasma Hg assessed by ICP-MS	Middle to late gestation (14th–40th week)	China	Case–control study	Total CHDs	Case: 303; control: 303	2.88 (1.22–6.77)	Maternal pre-pregnancy body mass index, education, occupation, parity, and periconceptional folic acid supplementation	(91)

As, arsenic; Ba, barium; BMI, body mass index; Cd, cadmium; Cr, chromium; Hg, mercury; ICP-MS, inductively coupled plasma mass spectrometry; NA, not available; Ni, nickel; Pb, lead.

**Table 4:** Summary of laboratory-based studies reporting the relationship between nonessential trace element exposure and CHDs.

Element	Animal model	Function	Potential mechanism	Reference
Cd	Zebrafish (*Danio rerio*) embryos cultured in Cd-containing media	Cu exposure led to hypertrophy of ventricle and pericardium; reduced heart rate; and abnormally elevated heart contractility	NA	(132)
Cd	Fertilized eggs injected with CdCl_2_ solutions	Cd exposure increased myocardial tissue area of the right ventricle	Cd exposure increased cell proliferation and upregulated expression of cell circle related genes in right ventricle	(133)
As	Zebrafish (*Danio rerio*) embryos cultured in As-containing media	As exposure decreased the amount of myocardium in ventricle, delayed cardiac looping, and dampened heart rate	NA	
As	Zebrafish (*Danio rerio*) embryos cultured in As-containing media	As exposure increased pericardial cavity as well as elongated atrium and ventricle	As exposure suppressed Dvr1 expression	(83)
As	Embryos from pregnant rats fed with As	As exposure increased risks of CHDs in offspring	As exposure increased Mef2C expression and H3K9 acetylation in fetal rat hearts	(84)
As	AV canal explants from chicken embryos were incubated on the collagen gel pretreated with sodium arsenite	As exposure perturbed EMT duringcardiac development	As suppressed TGF-β/Smad signaling	(86)
Ni	Marine medaka (*Oryzias melastigma*) embryos cultured in Ni-containing media	Ni exposure induced cardiovascular anomalies	Ni exposure led to dysregulated expression of cardiac development-related genes including *ATPase*, *smyd1*, *cox2*, and *bmp4*	(94)

ATPase, adenosine triphosphatases; As, arsenic; AV, atrioventricular; bmp4, bone morphogenetic protein 4; Cd, cadmium; cox2, cyclooxygenase 2; EMT, epithelial-mesenchymal transition; Ni, nickel; smyd1, SET And MYND domain containing 1; TGF-β, transforming growth factor β.

Zn, Cu, and Fe are essential trace elements for human body, however, both deficiency and excessive exposure of these elements may lead to health impairment (107, 108). Similar patterns could be seen when exploring association between these elements and CHDs. For instance, rodent embryos that deficient in Cu (26), Zn (58), or Fe (63) showed increased incidence of cardiac abnormalities. These results suggest that Zn, Cu, and Fe are pivotal elements in fetal cardiac development, and shortage of these elements may lead to CHDs. Conversely, several epidemiological studies and laboratory-based studies also revealed that excessive exposure to these trace elements increased risks of CHDs (28, 29, 38–41, 64). In addition, it should be noted that some epidemiological studies did not find the two-side effects of these trace elements in a single study, which may be attributed to: (i) the selected reference for comparison could not reflect the real population with deficiency or excessive exposure to certain trace element; (ii) did not analyze the non-linear dose–response effects between these elements and CHDs; (iii) or relatively small sample size leading to decreased statistical power.

Ferroptosis is a new form of regulated cell death characterized with Fe overload and lipid peroxidation, and may play pathogenetic roles in cardiovascular diseases (109). A recent study initially uncovered the potential role of ferroptosis in CHDs (110). In this study, gene silencing of T-box transcription factor 1 (TBX1), the candidate disease-causing gene of DiGeorge syndrome (also known as velo-cardio-facial syndrome), led to elevated ferroptosis in embryonic cardiomyocyte cell line H9c2. However, it is not clear whether inhibiting ferroptosis can reverse the phenotype of CHDs that induced by TBX1 deficiency; and whether Fe overload-induced ferroptosis is involved in the pathogenesis of CHDs. Recently, it has been found that Cu can directly bind to lipoylated components of the tricarboxylic acid cycle, leading to cell death in a Cu dependent manner, which may account for the toxicity of Cu overload (111). However, whether this newly form of regulated cell death is involved in CHDs that induced by high Cu exposure is also unknown. Studies concerning these filed may broaden our insights regarding association between essential trace elements and cardiac development.

The effects of trace elements on the human body are not completely independent, but may have synergistic, additive, or antagonistic effects. For instance, several essential trace elements such as Zn, Fe, and Se showed protective roles in Cd-induced toxicity (112). In addition, it has been shown that Pb and As have synergistic toxicity to the developing brain, leading to impaired neurobehavioral functions (113). The potential interactions between trace elements in CHDs have also been studied in several epidemiological studies, though most of the results were not significant (38, 64, 67, 114). Only a few studies in this field have obtained significant results. For instance, a synergistic interaction between maternal hair As and Cd were evident in CHDs by multivariate logistic regression model (74). These results are consistent with a previous study that showed synergistic toxicity of inorganic As and Cd on kidney (115). Moreover, a recent study conducted by Wang et al. explored the potential interactions between several selected metals in CHDs using a novel method of studying exposure-response function, i.e., Bayesian kernel machine regression (BKMR) (91). Significant interactions between Hg and Pb in CHDs were found in the same study. Similarly, other studies also found a synergistic interaction between Hg and Pb in impairing fetal growth and development (116, 117). Apart from the interactions between trace elements, the mixture effect of multiple elements in CHDs is also an intriguing issue that should be further addressed. Wang et al.’s used BKMR to investigate the association between maternal metal mixture exposure (Mn, Pb, Hg, Cd, and As in maternal plasma) and CHDs in offspring, and a significant positive association was observed (91). Moreover, Hg has been identified as the major contributor of the joint effect of metal mixture in the study (91). Besides BKMR, other novel methods such as weighted quantile sum (WQS) regression and quantile-based g-computation (QGcomp) have been proposed to evaluate the health effects of multi-pollutant mixture in epidemiological studies (118). Studies in this filed that utilize these models may provide a comprehensive insight on the roles of trace elements in CHDs in the real world.

In light of the importance of certain essential trace elements such as Cu, Zn, and Fe in cardiac development, it is meaningful to explore the potential benefit of trace element supplementation on CHDs prevention. Indeed, a previous study showed that dietary supplementation of Fe during mid-gestation in Fe deficient pregnant mice rescued the heart abnormalities in offspring (63). In support of the result, a case–control study found that mothers who delivering CHDs fetus were less likely to take Fe supplementation during pregnancy as compared with those delivering healthy fetus (27). The same group subsequently found that supplementation of Zn and Se but not Cu during pregnancy was associated with decreased risks of CHDs in offspring (47). Despite the encouraging results of these epidemiological studies, several limitation should be noted: (i) these studies were designed as retrospective observational studies, which may not provide establish causality and enough evidence for using trace element supplementation to prevent CHDs; (ii) the supplementation of trace elements was assessed through questionnaires, which may lead to potential recall bias; (iii) these studies did not explored the potentially different effects of trace element supplementation on pregnant women with stratified trace element status (i.e., sufficient, excessive or deficient trace element baseline levels). In addition, the effects of trace element supplementation on other adverse pregnancy outcomes such as preterm births, stillbirth, perinatal deaths, low birthweight, and other congenital anomalies should be taken into consideration comprehensively. Moreover, the potential adverse effects, even the possible teratogenicity of trace element supplementation during pregnancy should be carefully assessed in future.

In the last few decades, the accumulation of toxic heavy metals in soil, water, and air has become a growing environmental issue due to fossil fuel burning, the wild application of fertilizer and pesticide in agriculture, as well as the mining, smelting, and processing of metals (119). In particular, toxic heavy metal exposure during pregnancy has been closely associated with increased risks of gestational diabetes mellitus (120), preeclampsia (121), and several adverse pregnant outcomes such as spontaneous abortions, preterm births, and stillbirths (122). Most importantly, a few studies also found that maternal exposure to toxic heavy metals such as Cd, As, Pb, Ni, Ba, and Hg was associated with increased risks of CHDs in offspring, which further illustrates the essential for pregnant women to avoid toxic heavy metal exposure (74, 79–81, 91, 93, 100). Therefore, pregnant women are advised to (i) avoid the ingestion of food and water that were contaminated with heavy metals; (ii) avoid cigarette smoking, as it may be an important source of heavy metal exposure; (iii) avoid occupational exposure of heavy metals if possible (123, 124). Moreover, it has been found that melatonin, chelating agents, certain micronutrients, and several natural antidotes can rescue the toxicity of heavy metal exposure (125, 126). Further studies are warranted to investigate the potential application of these agents in pregnant women with high risks of heavy metal element exposure.

## Author contributions

YL, ZP, MZ, RG, YW, and YC responsible for literature research and writing. NZ reviewed the manuscript, made significant revisions on the drafts, and supervised and finalized this work. All authors have read and agreed to the published version of the manuscript.

## Funding

The research was supported by National Natural Science Foundation of China (nos. 81970738 and 81600157), Key Research and Development Program of Sichuan Province (no. 2020YFS0071), and Universal Application Program of Health Commission of Sichuan Province (no. 21PJ047).

## Conflict of interest

The authors declare that the research was conducted in the absence of any commercial or financial relationships that could be construed as a potential conflict of interest.

## Publisher’s note

All claims expressed in this article are solely those of the authors and do not necessarily represent those of their affiliated organizations, or those of the publisher, the editors and the reviewers. Any product that may be evaluated in this article, or claim that may be made by its manufacturer, is not guaranteed or endorsed by the publisher.
